# Adipokines in Liver Cirrhosis

**DOI:** 10.3390/ijms18071392

**Published:** 2017-06-29

**Authors:** Christa Buechler, Elisabeth M. Haberl, Lisa Rein-Fischboeck, Charalampos Aslanidis

**Affiliations:** 1 Department of Internal Medicine I, University Hospital Regensburg, 93042 Regensburg, Germany; elisabeth.haberl@klinik.uni-regensburg.de (E.M.H.); lisa.rein-fischboeck@klinik.uni-regensburg.de (L.R.-F.); 2 Institute of Clinical Chemistry and Laboratory Medicine, University Hospital Regensburg, 93042 Regensburg, Germany; charalampos.aslanidis@klinik.uni-regensburg.de

**Keywords:** adiponectin, leptin, MELD, ascites, portal vein

## Abstract

Liver fibrosis can progress to cirrhosis, which is considered a serious disease. The Child-Pugh score and the model of end-stage liver disease score have been established to assess residual liver function in patients with liver cirrhosis. The development of portal hypertension contributes to ascites, variceal bleeding and further complications in these patients. A transjugular intrahepatic portosystemic shunt (TIPS) is used to lower portal pressure, which represents a major improvement in the treatment of patients. Adipokines are proteins released from adipose tissue and modulate hepatic fibrogenesis. These proteins affect various biological processes that are involved in liver function, including angiogenesis, vasodilation, inflammation and deposition of extracellular matrix proteins. The best studied adipokines are adiponectin and leptin. Adiponectin protects against hepatic inflammation and fibrogenesis, and leptin functions as a profibrogenic factor. These and other adipokines are supposed to modulate disease severity in patients with liver cirrhosis. Consequently, circulating levels of these proteins have been analyzed to identify associations with parameters of hepatic function, portal hypertension and its associated complications in patients with liver cirrhosis. This review article briefly addresses the role of adipokines in hepatitis and liver fibrosis. Here, studies having analyzed these proteins in systemic blood in cirrhotic patients are listed to identify adipokines that are comparably changed in the different cohorts of patients with liver cirrhosis. Some studies measured these proteins in systemic, hepatic and portal vein blood or after TIPS to specify the tissues contributing to circulating levels of these proteins and the effect of portal hypertension, respectively.

## 1. Introduction

Liver cirrhosis is a severe disease whose underlying etiologies are mostly alcohol abuse, chronic hepatitis B virus (HBV) and hepatitis C virus (HCV) infections, as well as non-alcoholic steatohepatitis (NASH) [1]. NASH is the progressive form of non-alcoholic fatty liver disease (NAFLD), whose prevalence is steadily increasing because of the obesity epidemic [1,2].

High fat diets, Western-type diets, diets enriched in fructose and light to moderate alcohol intake could trigger the onset and progression of NAFLD [3,4,5]. Excess dietary fructose is connected to hepatic ATP depletion in type 2 diabetes patients and thus may contribute to NAFLD pathogenesis [6]. In patients with chronic HCV, daily intake of industrial fructose is linked to severe liver fibrosis [7].

Obesity per se raises the risk for decompensation of liver cirrhosis irrespective of the underlying etiology [8]. This is partly explained by the obesity-related rise of portal pressure [8]. Weight loss in overweight patients with liver cirrhosis though lowers portal pressure and possibly improves liver function [8,9].

Malnutrition and sarcopenia are further complications of liver cirrhosis patients and are relatively common in advanced disease [10]. Mortality risk, development of hepatic decompensation and secondary complications of liver cirrhosis are high in these patients [11,12].

Associations of obesity and malnutrition with severity of liver cirrhosis suggest that adipose tissue may have an impact on disease pathophysiology. Shifting lipid storage from subcutaneous to visceral fat tissues is well known to be associated with liver damages [13]. Functional differences of subcutaneous and visceral adipose tissue are associated with changes in their adipokine profiles [14,15] and possibly distinct levels of these adipokines in the portal and systemic blood [16]. We are, however, unaware of studies showing adverse effects of excess visceral fat mass on hepatic function in patients with liver cirrhosis.

Age of the patients seems to be also relevant for fibrosis progression. Elderly patients more likely have NASH and advanced liver fibrosis compared to younger NAFLD patients [17]. In older HCV-infected patients, an accelerated progression of fibrosis has been documented [18].

Gender affects the incidence and progression of liver diseases. Females are more susceptible to toxic insults like alcohol, but less often develop decompensated disease when infected with HCV [19,20]. Male gender is even a risk factor for NAFLD [21]. The likelihood of males to die from chronic liver disease or cirrhosis is two-fold higher compared to females [22].

The one-year mortality of patients with liver cirrhosis is 1% and may rise up to 57% in those with decompensated liver disease and complications [23]. The Child-Pugh or Child*-*Turcotte*-*Pugh score was established to predict risk in patients with liver cirrhosis upon insertion of a transjugular intrahepatic portosystemic shunt (TIPS) and is widely used to assess liver dysfunction. This score considers ascites, encephalopathy, total bilirubin, albumin and international normalized ratio (INR) [24,25]. The model for end-stage liver disease (MELD) score has been primarily developed to assess the survival of patients undergoing TIPS. The MELD score is calculated from total bilirubin, creatinine and INR values and is used for liver transplant priority ranking [24,26].

Serious complications of liver cirrhosis are ascites, variceal bleeding, hepatic encephalopathy and, most fatally, hepatorenal syndrome. Intrahepatic resistance and splanchnic vasodilation account for portal hypertension, which is the major cause of these sequelae [1,27]. Intestinal translocation of bacteria in advanced liver disease contributes to the hyperdynamic circulation [27,28] and spontaneous bacterial peritonitis, which may advance to sepsis and hepatic encephalopathy [1,27,28]. Liver cirrhosis of any cause is a risk factor for hepatocellular carcinoma, and increasing age, male gender, obesity and insulin resistance contribute to its development and progression [29].

Abnormal glucose metabolism is frequently diagnosed in liver cirrhosis [30]. Hyperinsulinemia, insulin resistance and raised hepatic glucose production are common in those patients [31]. Insulin resistance contributes to hepatic decompensation in the cirrhotic patient [32]. TIPS alleviate hepatic insulin clearance, thereby augmenting hyperinsulinemia, which may further worsen glucose homeostasis [33].

Body weight, fat free mass and muscle strength improve after TIPS [33,34]. Reversal of portal hypertension by TIPS is supposed to contribute to weight gain, but the underlying mechanisms are still unknown [34].

Adipokines are well known to regulate liver function. Circulating levels of adipokines are mostly altered in patients with liver cirrhosis and are partly related to MELD and/or Child-Pugh score. These associations are exciting and point to a functional role of adipokines in the pathophysiology of liver cirrhosis and its complications. The present review article summarizes studies having analyzed associations of systemic adipokine levels with the severity of liver cirrhosis.

## 2. Adipokines in Liver Cirrhosis

Various adipokines affect hepatic function, and only a few of them have been discussed in this review article. Adiponectin and leptin are the most studied adipokines so far, and various reports on their effects in the liver have been published [35,36]. Omentin, as well as adiponectin are reduced in obesity, while most, if not all, other adipokines are induced, and this indicates a specialized role of those proteins [2,37]. Galectin-3 is a well-described profibrotic factor and most likely contributes to liver injury in cirrhosis [38]. Interleukin-6 is an extensively analyzed cytokine and an acute phase protein regulating inflammatory responses [39,40]. Resistin seems to reflect macrophage activation in humans and, therefore, is supposed to be changed in liver cirrhosis [41]. Cirrhosis is a risk factor for hepatic carcinogenesis, which is blocked by chemerin. Altered levels of this chemokine in liver cirrhosis may predispose to tumor development [35,42]. Numerous studies have been published where systemic levels of these adipokines were analyzed in patients with liver cirrhosis to identify associations with disease severity and secondary complications, and we are unaware of a review article summarizing these results. The intention of the present work was to list these investigations to realize which of these proteins are concordantly changed in different cohorts of cirrhosis patients. Cross-sectional studies do not permit any functional conclusions, and preclinical studies on the role of adipokines in liver cirrhosis are sparse. Though we speculate about the pathophysiological function of these adipokines in liver cirrhosis, many issues are still unresolved.

### 2.1. Adiponectin

#### 2.1.1. General Information

**Figure 1:**
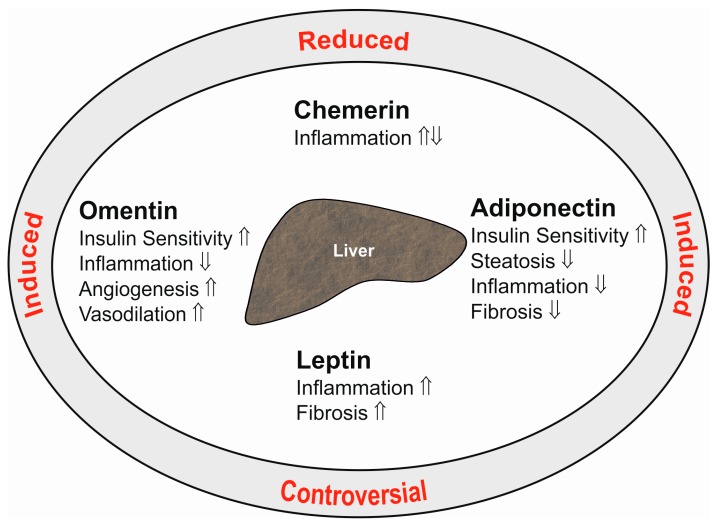
Summary of the hepatic effects of the adipokines adiponectin, leptin, chemerin and omentin in the liver (inner ellipse). ⇑ indicates a positive and ⇓ a negative effect on the respective pathways. The text at the outer ellipse tells whether systemic levels of these adipokines are induced or reduced in patients with liver cirrhosis compared to controls. “Controversial” indicates that contradictory findings have been published so far.

Adiponectin is a 28-kD protein almost exclusively secreted by adipocytes. High adiponectin levels circulate in serum and are reduced in obesity. Adiponectin ameliorates insulin sensitivity, and thus, lower serum levels are correlated with disturbed glucose metabolism. Adiponectin is an antisteatotic, anti-inflammatory and antifibrotic adipokine (Figure 1), and low levels may predispose to fatty liver and advanced hepatic injury [2].

Hepatic stellate cells are the main profibrotic cells in the liver. These cells become activated upon liver injury and start to proliferate and produce extracellular matrix proteins. Adiponectin reduces hepatic stellate cell activation, proliferation and survival [43,44]. Connective tissue growth factor is one of the profibrotic cytokines released by hepatocytes and is reduced by adiponectin [45]. Beneficial activities of this adipokine in the liver have been thoroughly summarized in recent review articles, which are recommended for further reading [2,46,47,48].

Because it is not feasible to raise circulating adiponectin in humans, present investigations test the suitability of adiponectin analogues. These agents attenuate liver fibrosis in animal models [49,50]. Thioacetamide-induced activation of hepatic stellate cells and liver resident macrophages are markedly suppressed, suggesting that these molecules have the potential to become new anti-fibrotic therapeutics [50].

Adiponectin receptors 1 and 2 are expressed in most cells and tissues, and ceramidase becomes activated upon binding of adiponectin [51,52]. Downstream targets of adiponectin, which may be activated by metabolites derived from ceramidase activity, are adenosine monophosphate -activated protein kinase and peroxisome proliferator-activated receptor α [51,52,53]. Both are well characterized concerning their hepatoprotective activities [54,55].

Adiponectin is cleared from the circulation primarily by the liver. Female humans and rodents have higher plasma adiponectin levels than males. Clearance rate is, however, comparable in both sexes, suggesting that systemic levels are defined by adiponectin production in fat tissues [56].

Different adiponectin multimers circulate in serum. Trimers, hexamers and high molecular weight (HMW) forms have been identified, with beneficial effects in metabolism being attributed to the latter form [2].

#### 2.1.2. Circulating Adiponectin in Liver Cirrhosis

Various studies have analyzed whether systemic adiponectin is changed in patients with liver cirrhosis when compared to patients with chronic liver disease or healthy-liver controls (Table 1). Increased systemic adiponectin in the cirrhotic patients has been identified in all of the studies (Table 1) [57,58,59,60,61,62,63]. Importantly, elevated adiponectin is found in the patients independent of disease etiology (Table 1) [57,58,59,60,61,62,63,64,65]. Higher adiponectin was identified in normal-weight and overweight patients independent of the age of the participants in each cohort (Table 1). Interestingly, there are no associations of serum adiponectin with the homeostasis model assessment (HOMA) index, as a surrogate marker of insulin sensitivity [59,61] or body mass index (BMI) [63,65] in cirrhosis patients. High adiponectin even increases the risk to develop hepatocellular carcinoma in HCV-infected patients [66] suggesting that the well-characterized hepatoprotective and anti-carcinogenic effects of this adipokine are blocked [2,67].

In rats with liver fibrosis, HMW adiponectin is the only isoform found increased in serum [68]. In HCV-infected patients, total and HMW adiponectin are higher in cirrhosis [64,66]. Here, total, but not HMW serum adiponectin levels positively correlate with histologically scored liver fibrosis and inflammation [64]. Additional studies are required to elucidate the distribution of adiponectin isoforms in liver cirrhosis patients.

Elevated serum adiponectin is also found in patients with rheumatoid arthritis, inflammatory bowel disease and type 1 diabetes [69,70,71]. Impaired elimination from the circulation, higher release from fat tissues eventually stimulated by disease-associated hormones or drugs have all been discussed to contribute to raised serum adiponectin [70]. Up to now, neither the source, nor the pathophysiological role of elevated adiponectin have been unraveled.

Only one study has compared adiponectin in patients with non-cirrhotic and cirrhotic chronic liver disease and demonstrates increased levels in the latter group [72] (Table 1). Adiponectin is further positively associated with Child-Pugh stage and/or MELD score [57,58,59,60,63,65] (Table 1). This principally indicates that high adiponectin is indeed related to liver cirrhosis and further suggests that the liver has a central function in the excretion of adiponectin.

Similar concentrations of adiponectin in a peripheral vein, the portal vein and the hepatic vein do not support this suggestion [59,74] (Table 2). However, when also considering the blood flow, hepatic adiponectin extraction declines in parallel with the clinical stage of liver cirrhosis [63]. Bile duct ligation in mice increases serum adiponectin, and this may further point to biliary excretion of this protein [72,75]. In cholestatic patients with liver cirrhosis, higher and unchanged adiponectin is found compared to cirrhotic controls [61,72]. Adiponectin is nevertheless detected at high levels in human bile [72], indicating that the impaired biliary excretion route in liver cirrhosis patients contributes to its raised serum levels.

Body cell mass defined by bio-electrical impedance analysis is a measure of the metabolically-active body tissues [34]. TIPS placement leads to an increase of body cell mass and serum adiponectin. Whether raised adiponectin is indicative for worsened hepatic clearance after TIPS needs further study [76].

Esophageal varices are common complications in liver cirrhosis and are diagnosed by endoscopic techniques. Non-invasive biomarkers to predict varices may help to reduce the number of screening endoscopies [77]. A score calculated from platelet count, HOMA-insulin resistance (HOMA-IR) and adiponectin predicts esophageal varices with a high sensitivity and specificity [78]. Whether this approach is suitable in clinical practice needs further analysis.

Higher adiponectin in patients with liver cirrhosis and ascites compared to those without ascites has been described in some [60,65], but not all studies [61]. Ascites mostly develops in patients with more advanced liver dysfunction [65], and this may partly explain raised adiponectin in those patients. Serum adiponectin is about five-fold higher when compared to levels in ascites [79]. This excludes excess adiponectin production in ascites to contribute to serum levels.

Though adiponectin is markedly elevated in cirrhosis and positively correlates with MELD and Child-Pugh score, it is not a suitable biomarker to predict overall survival [65].

Circulating adiponectin is increased in patients with liver cirrhosis independent of disease etiology, age or BMI. Most studies have identified positive associations with Child-Pugh or MELD score and impaired biliary excretion of adiponectin may be one pathway contributing to this correlation. Increased adiponectin in liver cirrhosis does not seem to exert any protective effects. This may be because of downregulation of the respective receptors in the cirrhotic liver [75,80]. In advanced NASH, a negative association of serum adiponectin and liver fat content has been identified, suggesting that this adipokine contributes to hepatic lipid waste [81]. Adiponectin may, therefore, also have a role in fat loss and hypermetabolism in liver cirrhosis [82].

### 2.2. Chemerin

#### 2.2.1. General Information

Chemerin is an attractant for immune cells and is highly expressed in adipocytes and hepatocytes [87,88,89]. Chemerin is released by the cells as an inactive (18 kD) proprotein. Carboxy-terminal processing by proteases produces isoforms that lack six (Chem157), eight (Chem155) or nine (Chem154) amino acids. Among these truncated proteins, Chem157 is most active in recruiting immune cells [90]. Chem157 is commercially available as a recombinant protein and is the best studied isoform. The biologic function of additional chemerin variants has not been clarified yet [87]. Chemerin may act as a pro- or anti-inflammatory mediator dependent on the equilibrium of its different isoforms [87,90,91] (Figure 1). Receptors for chemerin are chemokine-like receptor 1 (CMKLR1), G-protein coupled receptor 1 (GPR1) and CC-motif chemokine receptor-like 2 (CCRL2). The latter is a non-signaling molecule that binds chemerin to increase its local bioactivity [90].

Chemerin and its receptor CMKLR1 are expressed in hepatocytes and hepatic stellate cells [89,92]. Though chemerin may exert autocrine and/or paracrine activities in the liver, its function in this organ is still unclear. Indeed, chemerin, GPR1 and CMKLR1 knock-out mice do not display gross hepatic abnormalities even when fed diets to produce obesity or NAFLD [93,94,95]. Chemerin and GPR1 seem to stimulate insulin secretion and knock-out animals consequently display hyperglycemia [94,95]. One study has shown that hepatic overexpression of chemerin in mice impairs skeletal muscle, but not hepatic insulin response [96].

Circulating chemerin is raised in obesity, which is mediated by higher synthesis in fat tissues [87,97]. The bulk of human studies indicate associations of chemerin with the metabolic syndrome [87,91,98,99]. Indeed, positive associations of circulating chemerin with BMI, fat mass and markers of insulin resistance have been identified [87]. Of note, while total chemerin serum levels are raised in obesity, the portion capable of activating CMKLR1 is unchanged. The underlying mechanisms have not been resolved yet, and enhanced removal of active chemerin isoforms and/or impaired processing of prochemerin may be involved [100]. In serum of obese humans, relatively short chemerin isoforms have been recently detected. Whether these short forms are derived from chemerin degradation and exert any biologic activity is not well investigated [101].

Measurement of chemerin in murine and human body fluids by enzyme-linked immunosorbent assays does not distinguish the different chemerin isoforms, though their distribution may be changed in the patients [100].

Serum chemerin is raised in HCV infected patients, but is not related to HOMA-IR or liver fibrosis [102]. In NAFLD patients, both higher and unchanged serum chemerin levels have been reported [91]. This principally excludes a strong association of chemerin in serum and hepatic function.

#### 2.2.2. Circulating Chemerin in Liver Cirrhosis

To the best of our knowledge, there are only two studies having analyzed systemic chemerin in liver cirrhosis patients (Table 3). In one study, patients with mostly HCV-induced cirrhosis and hepatocellular carcinoma were enrolled. The second study included patients with mainly alcoholic liver cirrhosis [83,103].

The obesity and diabetes related parameters BMI, fasting plasma glucose levels, fasting insulin levels, hemoglobin A1c and HOMA-IR do not correlate with chemerin in serum [103]. Both studies describe a negative association of serum chemerin with the Child-Pugh score [83,103]. Chemerin in serum does not correlate with the MELD score, albumin, bilirubin, aspartate aminotransferase, alanine aminotransferase and fibrinogen in one study [83]. The second analysis identified positive correlations with platelet counts and albumin and negative associations with bilirubin and alanine aminotransferase [103]. The two studies agree that serum chemerin is positively associated with prothrombin time [83,103]. This suggests that serum chemerin is related to coagulation rather than liver function in the patients. Indeed, chemerin is similar in patients with compensated and decompensated liver cirrhosis in a subgroup of patients with normal prothrombin time [83].

Serum chemerin is not related to variceal size or ascites. Portal vein chemerin is nevertheless higher in patients with ascites. This may resemble elevated release from omental fat depots and/or reduced hepatic uptake [83].

In the liver, hepatocytes are the main producers of chemerin. Protein is also released by hepatic stellate cells [89]. Hepatic vein chemerin in liver cirrhosis is higher than portal vein concentrations, demonstrating enhanced synthesis and secretion by the liver cells [83] (Table 2).

The function of chemerin in the liver has not been studied in more detail. Chemerin inhibits the growth of liver tumors [42], and prospective studies may identify an association of low serum chemerin and the development of hepatocellular carcinoma. Future experiments should focus on the physiological and pathophysiological roles of chemerin isoforms in the circulation and the liver.

### 2.3. Leptin

#### 2.3.1. General Information

Leptin is a 16-kD hormone that regulates satiety and energy expenditure. Mutations in the leptin gene or the leptin receptor gene contribute to obesity. Serum leptin is nevertheless raised in the obese who display leptin resistance in the hypothalamus [104]. Pleiotropic effects of leptin have been identified. This hormone regulates the immune system, hematopoiesis, angiogenesis, reproduction and carcinogenesis among others [35,105,106].

Moreover, leptin is known to contribute to fibrogenesis in chronic liver diseases. Leptin is a mitogen for activated hepatic stellate cells and further enhances the synthesis of inflammatory and profibrogenic factors in these cells. It increases transforming growth factor β synthesis in Kupffer cells, which promotes fibrosis progression. Activated hepatic stellate cells are capable of producing leptin, and this is supposed to augment hepatic damage [35,107,108,109] (Figure 1).

Various review articles have been published summarizing the inflammatory and profibrogenic effects of leptin in the liver [44,46,110]. Therefore, these issues are not further addressed herein.

#### 2.3.2. Circulating Leptin in Liver Cirrhosis

Systemic leptin is higher in healthy females, and this also applies for patients with liver cirrhosis [111,112]. Gender-related differences were not described in all studies or were simply not calculated because of the small number of patients enrolled or the similar distribution of males and females in the respective cohorts [113,114,115]. Though some investigations show comparable changes of leptin levels in both sexes, other investigations have identified gender-related differences [60,112]. In one study, leptin is only raised in female cirrhotic patients, while a separate analysis has found decreased levels in female, but not male patients [60,112] (Table 4).

Several studies report that leptin is increased in patients with liver cirrhosis [111,112,116]. Separate investigations have shown reduced leptin levels in the cirrhotic patients [60,113]. Further, unchanged circulating leptin in cirrhotic patients has been also described [73,114]. Discordant findings are not related to disease etiology (Table 4). It is important to note that all of the studies listed in Table 4 have enrolled healthy controls. Thus, it is not clear whether leptin is further changed in liver cirrhosis when compared to non-cirrhotic patients with chronic liver diseases.

Though positive associations of leptin with HOMA-IR have been identified [117], this adipokine does not correlate with blood glucose and insulin [114]. Accordingly, impaired glucose tolerance and type 2 diabetes are not linked to changes in leptin levels [118].

Systemic leptin positively correlates with BMI in patients with liver cirrhosis [114,115,117]. Malnourished cirrhotic patients have lower serum leptin than appropriately-nourished patients [117]. This suggests that anthropometric variables and nutritional status are closely associated with serum leptin and have to be considered when analyzing systemic leptin levels in patients with liver cirrhosis.

Several studies investigated whether circulating leptin levels are correlated with severity of liver dysfunction. Here, raised leptin has been identified in male patients with a higher Child-Pugh score [119]. A separate study describes a transient increase of serum leptin in early stages of liver disease, which normalize in those patients with advanced disease [120]. No associations of leptin and Child-Pugh score [111,114] and lower levels in patients with hepatic decompensation [118] have been also reported (Table 4).

Discordant findings on leptin levels in liver cirrhosis patients indicate that this adipokine is closely linked to BMI and nutritional state rather than hepatic function. Indeed, after adjusting for BMI, serum leptin is not associated with disease severity in non-diabetic patients with alcoholic liver cirrhosis [65]. A prospective, multicenter, uncontrolled pilot study enrolled patients with a BMI ≥ 26 kg/m^2^, compensated liver cirrhosis and portal hypertension. The intensive 16-week lifestyle intervention program decreased body weight, portal hypertension and systemic leptin levels. Child-Pugh scores and MELD scores were not improved, arguing against a close relationship between systemic leptin levels and clinically-scored disease activity [8].

Leptin is neither changed in patients with ascites, nor in those with esophageal varices [60,65,78,119]. A relation of circulating leptin with hepatic encephalopathy, cholestasis and renal disease has been excluded [60].

The cytokines interleukin-6 and -8 (IL-6, IL-8) and TNF in ascites are above serum levels [121]. Leptin is nearly two-fold more concentrated in ascites than in serum [74,122]. Positive correlations of serum and ascites leptin levels indicate that ascites leptin contributes to systemic levels [74,122]. Though patients with liver cirrhosis and ascites have higher IL-6 and IL-8 in serum than those without ascites [121], serum leptin is unchanged [65,119]. To what extent proteins found at high concentrations in ascites do add to serum levels is not exactly known.

Subcutaneous fat releases two- to three-fold more leptin than visceral adipose tissues [123]. Accordingly, plasma leptin concentration is higher in the radial artery than the portal vein in very obese subjects [16] and in patients with liver cirrhosis [74] (Table 2). Subcutaneous adipose tissue is the major source of serum leptin, and this seems to also apply for patients with liver cirrhosis. Whether leptin release from fat depots is changed in the patients or is even raised in intraabdominal adipose tissues of those patients with ascites needs further clarification.

TIPS contribute to weight gain, and leptin may simultaneously increase. Higher body weight after this intervention is, however, not consistently accompanied by raised leptin levels [76,124].

Altogether, serum leptin is mostly not associated with liver dysfunction. Compared to the healthy liver controls, its levels are higher, lower or normal in patients with liver cirrhosis. Discordant findings may be due to the close association of serum leptin with BMI and body fat mass.

### 2.4. Omentin

#### 2.4.1. General Information

Omentin is a 30-kD protein, which is expressed by stromal-vascular cells in adipose tissue. Interestingly, omentin is highly abundant in visceral fat [125]. In obesity, expression of omentin declines in this adipose tissue depot, and circulating omentin is reduced [37,125]. Omentin is an anti-inflammatory protein and further improves insulin sensitivity [125,126] (Figure 1). Whether omentin directly regulates the biologic function of liver cells has not been clarified yet.

Omentin induces vasodilation and angiogenesis partly by activating endothelial nitric oxide synthase [127,128]. Inappropriate levels of nitric oxide contribute to splanchnic vasodilation and hepatic vasoconstriction and consequently portal hypertension [129]. Portal hypertension leads to the serious complications of liver cirrhosis, such as ascites, bleeding from varices, encephalopathy and renal dysfunction [130].

#### 2.4.2. Circulating Omentin in Liver Cirrhosis

Omentin levels tend to be higher in patients with liver cirrhosis compared to healthy-liver controls [84] (Table 5). In HCV-infected patients, serum omentin is increased. Neither hepatic omentin mRNA, nor its serum levels are associated with features of liver injury [131]. No differences in omentin serum levels regarding the severity of liver cirrhosis evaluated by the Child-Pugh or MELD scores have been identified [84,132] (Table 5). There are no changes in patients with ascites or varices [84].

In agreement with its preferred synthesis in intra-abdominal fat depots, portal vein omentin is higher than the hepatic vein level in healthy-liver controls and cirrhotic patients [84,125] (Table 2). Of note, omentin is induced in the portal vein of the patients compared to the controls, and this may reflect raised release from intraabdominal fat and/or impaired hepatic clearance. Portal vein omentin does neither correlate with hepatic venous pressure gradient, nor complications of portal hypertension [84].

Circulating omentin in liver cirrhosis has been analyzed in two relatively small cohorts of patients so far. Both studies agree that omentin is not associated with severity of liver cirrhosis.

### 2.5. Galectin-3

#### 2.5.1. General Information

**Figure 2:**
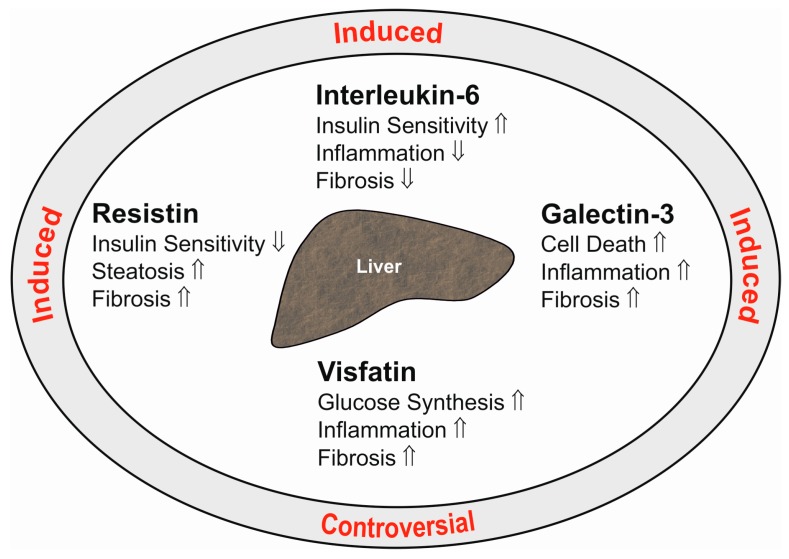
This figure summarizes the hepatic effects of the adipokines resistin, interleukin-6, galectin-3 and visfatin in the liver (inner ellipse). ⇑ indicates a positive and ⇓ a negative effect on the respective pathways. The text at the outer ellipse is related to the respective levels in serum of patients with liver cirrhosis. “Controversial” means that contradictory findings have been published so far.

Galectin-3 is a 30-kD protein expressed by various cells including immune cells and adipocytes [133,134]. Visceral human fat displays higher galectin-3 levels than subcutaneous adipose tissue. Hepatic galectin-3 is induced in obesity [134], and systemic galectin-3 is increased [135]. Galectin-3 exerts various biological functions and regulates cell death, inflammation, angiogenesis and collagen synthesis (Figure 2). Blockage of galectin-3 prevents activation of hepatic stellate cells and, accordingly, expression of collagen. Loss of galectin-3 protects mice from fibrosis due to carbon tetrachloride [136]. Hepatic formation of advanced lipoxidation end products contributes to inflammation and fibrosis in NASH [137]. Galectin-3 mediates the uptake of these particles into the liver, and mice deficient in galectin-3 are protected from hepatitis and fibrosis in high fat diet models [136,138].

Macrophages and cholangiocytes express galectin-3 in the healthy liver. In cirrhotic liver, galectin-3 is further detected in hepatocytes [85,139], suggesting raised hepatic galectin-3 production in liver cirrhosis.

In a model of thioacetamide-induced liver injury, in murine NASH and in NASH patients, inhibition of galectin-3 improves hepatic inflammation, cell death and fibrosis [38,133,140].

#### 2.5.2. Circulating Galectin-3 in Liver Cirrhosis

Serum galectin-3 is increased in patients with liver cirrhosis compared to the healthy controls and non-cirrhotic patients with chronic liver disease [85,141,142] (Table 6). Levels are higher in the patients with alcoholic cirrhosis than in patients with non-alcoholic cirrhosis mostly due to HCV [141]. In a separate study, HCV-infected patients had higher galectin-3 than patients with alcoholic or cryptogenic cirrhosis [85]. A third study compared HBV- and HCV-caused cirrhosis and described increased galectin-3 in the latter cohort [142].

Galectin-3 is positively associated with the Child-Pugh score, and levels are highest in patients classified as C [85,141]. Positive correlations of galectin-3 and MELD score have been described in patients with alcoholic liver cirrhosis [85] (Table 6). Galectin-3 is already elevated in patients with little ascites, but does not further increase in parallel with ascites volume [85].

In healthy-liver controls, galectin-3 is higher in portal venous serum compared to hepatic venous serum and systemic levels, which is principally in line with higher expression in intra-abdominal fat depots [135]. In liver cirrhosis patients, this distribution is changed, and galectin-3 concentrations are higher in the hepatic vein than the portal vein blood [85] (Table 2). This is in accordance with raised hepatic expression of galectin-3 in the fibrotic liver [85].

Galectin-3 in serum is increased in liver cirrhosis and is positively associated with Child-Pugh and MELD scores. Levels may be further affected by the etiology of liver cirrhosis, but this needs further analysis. Galectin-3 is a profibrotic protein and, thus, most likely contributes to liver injury in patients with cirrhosis [38].

### 2.6. Resistin

#### 2.6.1. General Information

Resistin is a 12-kD protein and has been discovered as an adipokine in rodents. In the animals’ serum, resistin is consistently higher in obesity, and there is a clear relationship between resistin levels and insulin resistance. In mice fed a high fat diet, normalization of resistin levels by antisense oligonucleotides reverses hepatic insulin resistance [41,143].

In humans, macrophages express and release this protein, and these cells are supposed to be the major source of serum resistin [41].

Adipokines that are expressed and secreted in higher quantity from visceral fat may be increased in the portal vein when compared to systemic levels [16]. These proteins are supposed to contribute to the metabolically-harmful effects of visceral adiposity [14]. Resistin is not raised in the portal vein, arguing against higher release from macrophages in intra-abdominal adipose tissues of humans [16].

Conflicting results regarding elevated systemic resistin levels in human obesity and associations with insulin resistance have been published [41]. Case-control studies have nevertheless shown that baseline serum resistin levels are associated with the risk to develop type 2 diabetes [144].

Liver macrophages are activated upon hepatic injury and are involved in the development of inflammation and fibrosis [145]. Resistin expression is indeed raised in the fibrotic human liver [146]. In human NASH liver, Kupffer cells and hepatic stellate cells express resistin, which also predicts fibrosis severity [147]. In hepatic stellate cells, resistin is shown to induce monocyte chemoattractant protein-1 and IL-8 [146]. This protein further mediates proliferation of these cells and suppresses apoptotic cell death [148]. Resistin upregulates the class A scavenger receptor in macrophages, which contributes to uncontrolled lipid uptake [149]. Downregulation of the hepatic low-density lipoprotein (LDL) receptor [150] and stimulation of de novo lipogenesis in liver cells by resistin may further contribute to dyslipidemia and liver steatosis [150]. This may add to insulin resistance, which has been recently identified in resistin-incubated HepG2 cells [151] (Figure 2).

#### 2.6.2. Circulating Resistin in Liver Cirrhosis

Resistin is found increased in patients with liver cirrhosis even when compared to non-cirrhotic patients with chronic liver injury. This is described in cirrhosis due to alcohol and viral infections [58,60,74,152,153] (Table 7). Bahr et al. report that resistin is lower in liver cirrhosis caused by viral infection when compared to alcoholic- and biliary-induced cirrhosis [152].

Resistin serum levels are positively associated with the Child-Pugh score in some, but not all studies [58,60,74,152,153] (Table 7). Indeed, hepatic vein resistin is higher in Child-Pugh C than A patients, suggesting increased secretion from the liver [74].

One of the studies could not demonstrate an association of resistin with liver dysfunction scores, but here, resistin was higher in patients with cholestasis and patients with renal impairment [60].

Data on the association of systemic resistin with insulin resistance in patients with liver cirrhosis are inconsistent [58,152].

Resistin is mostly expressed in macrophages and, accordingly, positively correlates with inflammatory and profibrotic cytokines produced by these cells [154].

Resistin is not associated with portal hypertension and is not changed in patients with ascites [65,152]. Ascites’ resistin is about two-fold higher when compared to systemic levels [79], demonstrating that resistin production is raised in the peritoneal cavity.

In liver cirrhosis, resistin concentrations are comparable in the portal, hepatic and systemic blood [74] (Table 2). This principally excludes that its synthesis is selectively induced in visceral fat, the spleen, intestine or the liver.

Macrophage activation is a common feature of liver cirrhosis, which may be reflected by a rise in serum resistin. This protein contributes to hepatic inflammation and fibrosis, indicating harmful effects of higher circulating levels in liver cirrhosis [146,148].

### 2.7. Visfatin

#### 2.7.1. General Information

Visfatin (nicotinamide phosphoribosyl-transferase, pre-B cell colony-enhancing factor) has a molecular weight of about 55 kD and is a key enzyme in cellular nicotinamide adenine dinucleotide biosynthesis. Cellular visfatin is low in NAFLD, thereby contributing to hepatocyte apoptosis [155].

Secreted visfatin functions as a multifunctional adipokine. Visfatin expression is induced in adipose tissues of obese patients, and its systemic levels are increased in obesity [156]. Visceral and subcutaneous adipose tissues express similar levels of visfatin [156,157]. Accordingly, portal vein, hepatic vein and systemic visfatin concentrations are equal in patients with normal liver function [74]. Hepatocytes constitutively release visfatin [158], but these concentrations fail to markedly increase levels in the hepatic vein [74].

The functions of secreted visfatin are manifold [159]. In hepatocytes, visfatin is shown to upregulate glucose production [160]. Cultivation of hepatocytes in the presence of palmitate is used as a model for liver steatosis. These cells release IL-6 and TNF in high quantity, and this is further induced by visfatin [161] (Figure 2). In hepatic stellate cells synthesis of α‑smooth muscle actin, collagens and connective tissue growth factor are induced by visfatin, indicating profibrotic activity of this cytokine [162] (Figure 2). In line with this in vitro study, an in vivo analysis shows reduced hepatic collagen and α‑smooth muscle actin upon suppression of hepatic visfatin in tetracarbon chloride and ethanol-treated rodents [163].

#### 2.7.2. Circulating Visfatin in Liver Cirrhosis

One study has described reduced systemic and hepatic visfatin protein in patients with cirrhosis when compared to healthy controls [164]. Strongly diminished visfatin in the circulation has been also identified in a second analysis [74]. Separate studies in HBV-related cirrhosis show higher circulating visfatin levels in patients with liver cirrhosis in relation to healthy controls and non-cirrhotic HBV-infected patients [165,166] (Table 8).

One study shows positive correlations with the Child-Pugh score, which has not been described in further cohorts [65,74,164,165,166] (Table 8). Portal vein and hepatic vein visfatin are higher when compared to systemic levels [74] (Table 2). This may indicate elevated release of visfatin from omental fat and eventually the liver in cirrhotic patients. In consideration that low hepatic visfatin protein has been detected in the cirrhotic liver [164], this issue is far from being well understood.

Visfatin in serum is not changed in those patients who develop ascites [65,79], and ascitic visfatin levels are not related to the Child-Pugh score [79].

So far, only a few studies have reported on systemic visfatin in patients with liver cirrhosis, and moreover, the data are conflicting.

### 2.8. Interleukin-6

#### 2.8.1. General Information

Interleukin-6 (IL-6) is a 21-kD cytokine that is associated with inflammation. IL-6 is produced by various cell types including adipocytes and immune cells [16,167]. Serum IL-6 is raised after infections and in most inflammatory disorders including chronic liver diseases [167]. Chronically-increased IL-6 contributes to inflammation and suppresses insulin signaling [168]. On the other hand, IL-6 signaling specifically in hepatocytes protects the animals from hepatic inflammation and insulin resistance [169] (Figure 2). Hepatic IL-6 signaling induces the acute-phase response, which counteracts bacterial infection and liver injury [40].

In a preclinical model of liver disease, mice pretreated with IL-6 have less hepatic injury [170]. Accordingly, hepatic dysfunction and hepatocyte death are aggravated in IL-6-deficient mice [171]. Loss of the IL-6 signal transducing molecule gp130 in non-parenchymal liver cells increases liver fibrogenesis, while its deletion in hepatocytes is without any effect [40]. Lack of this receptor is linked to pronounced bacterial growth after bile duct ligation and a higher mortality [172].

Acute and chronic raise in IL-6 may exert opposing biologic activities. While short-time elevation is mainly protective, chronically-increased levels are mostly related to harmful effects [36]. C-reactive protein is one of the factors induced by IL-6 signaling in hepatocytes [16,173]. Portal vein IL-6 is about 50% higher than levels in the radial artery and is positively correlated with serum CRP [16]. This clearly shows that IL-6 released by intra-abdominal adipose tissue is directly related to systemic inflammation in the obese.

#### 2.8.2. Circulating IL-6 in Liver Cirrhosis

Basically, most of the studies show increased systemic IL-6 in patients with liver cirrhosis [39,40,153,174,175] (Table 9). Healthy probands and non-cirrhotic patients with chronic liver disease served as controls. IL-6 levels are higher in alcoholic liver cirrhosis than in HCV or cryptogenic cirrhosis [86,176,177] (Table 9). Ethanol increases gut permeability and bacterial translocation [178]. Monocytes of patients with alcoholic cirrhosis display an exacerbated response to lipopolysaccharide, which is not observed in HCV-infected patients [179,180]. Thus, increased release of this cytokine by peripheral and tissue resident macrophages seems to contribute to higher levels in alcoholic cirrhosis.

Further, serum IL-6 levels increase with a higher Child-Pugh score [39,40,86,175] (Table 9).

Analysis by immunohistochemistry failed to detect IL-6 in the cirrhotic liver, thus excluding a possibly raised hepatic production as the cause for induced serum levels [39]. IL-6 is markedly lower in the hepatic compared to the portal vein of healthy-liver controls and is also reduced in the patients albeit to a lesser extent (Table 2). Therefore, impaired removal by the cirrhotic liver may constitute one mechanism contributing to higher systemic levels [16]. Indeed, only 6.3% of portal vein IL-6 is cleared by the cirrhotic liver, while about 40% is removed by the healthy liver [86]. Hepatic removal of IL-6 declines with increasing Child-Pugh score, and this may contribute to positive associations of systemic IL-6 levels and liver dysfunction [39,40,86,175] (Table 9).

IL-6 is further increased in serum of patients with ascites [86,121]. It is about 12-fold higher in ascites than in serum, demonstrating strongly induced production in the peritoneal cavity [121], which may partly contribute to higher circulating levels in those patients.

Portal hypertension leads to ascites and is positively correlated with serum IL-6. Hepatic IL-6 removal is not associated with the hepatic venous pressure gradient, and lowering of portal hypertension by TIPS has no effect on systemic IL-6 levels [86,176]. Therefore, portal hypertension does not affect IL-6 levels in patients with ascites.

A separate study has analyzed whether IL-6 is linked to hepatic encephalopathy. The authors have identified positive correlations of serum IL-6 with plasma ammonia and severity of overt hepatic encephalopathy [181].

Impaired uptake of IL-6 by the liver is linked to inappropriate IL-6 signaling and, thus, poor acute phase response in these patients [170,176]. Low expression of the hepatic IL-6 receptor and high circulating soluble gp130 further disturb IL-6 signaling [176].

Though IL-6 is supposed to contribute to hepatic insulin resistance, its levels are similar in cirrhotic patients with normal and high HOMA-IR [174,182].

High IL-6 in patients with liver cirrhosis is partly due to impaired hepatic clearance. This contributes to low hepatic IL-6 signaling and a poor acute phase response.

## 3. Conclusions

Systemic levels of adiponectin, omentin, resistin, galectin-3 and IL-6 are higher in patients with liver cirrhosis, while chemerin is reduced. Concentrations of these adipokines are partly correlated with residual liver function. Whether there are any causal relationships between changed adipokine levels and worse liver function cannot be evaluated in cross-sectional studies. Though adiponectin has been described as a protective adipokine, current evidence does not support a beneficial function in critically ill patients. Biologic activity of systemic adipokines not only depends on total concentrations, but is also affected by receptor levels, the formation of multimers in the case of adiponectin and proteolysis in the case of chemerin [2,87,185]. The pathophysiological relevance of changed systemic adipokines in liver cirrhosis patients has not been clarified yet.

## 4. Perspectives

Though numerous studies have analyzed circulating adipokines in liver cirrhosis, their pathophysiological role is still unclear. Adipokines are released from adipose tissues and contribute to disease severity. Excess adipose tissue may accelerate disease progression, while its loss in advanced disease is also harmful. Whether fat depot distribution has an effect herein has not been analyzed in more detail yet. Data on circulating visfatin in liver cirrhosis are not concordant, and further studies are needed to clarify whether its levels are altered in the disease. Further, most studies have compared systemic adipokines in patients with liver cirrhosis and healthy controls. Here, appropriate patients with non-cirrhotic liver have to be enrolled to confirm that changed adipokine levels are indeed related to liver cirrhosis and not to the underlying disease. Experiments in preclinical models are needed to resolve the function of altered adipokines in liver cirrhosis. Prospective studies in human cohorts may be performed in the future to identify functional associations in the patients.

## Figures and Tables

**Table 1:** Adiponectin levels in ten different cohorts of patients with liver cirrhosis. Cohort size, % males, age, body mass index (BMI) and disease etiology are listed. Numbers are given as the mean ± standard deviation or standard errors of the mean (SEM), which is indicated. Data are also presented as median and range (according to the respective publications cited). ↑ indicates that systemic levels are higher in the liver cirrhosis patients than the healthy controls (HC) or patients with chronic liver disease (CLD). Table also lists the associations of serum adiponectin with the Child-Pugh score and/or model for end-stage liver disease (MELD) score. n.d., not described; HCV, hepatitis C virus.

Number of Patients (% Male)	Age (Years)	BMI (kg/m^2^)	Etiology	Systemic Levels	Positive Associations with Scores	Reference
147 (73)	49.6 ± 11.9	n.d.	Alcohol	↑ HC	MELD, Child-Pugh	[60]
45 (64)	57	28.1	Mixed	↑ HC	n.d.	[57]
38 (68)	63 ± 15.5	21.9 ± 2.7	Mixed	↑ HC	Child-Pugh	[62]
87 (51)	n.d.	n.d.	Mixed	↑ HC	Child-Pugh	[59]
40 (75)	53.3 ± 8.2	27.9 ± 4.3	HCV	↑ HC	n.d.	[61]
20 (75)	47.6 ± 2.4 (SEM)	23.0 ± 0.6 (SEM)	Mixed	↑ HC	Child-Pugh	[63]
79 (60)	66.6 ± 8.9	22.3 ± 2.9	HCV	↑ HC	Child-Pugh	[58]
40 (88)	59 (37–75)	25.89 (20.1–39.3)	Alcohol	n.d.	Child-Pugh	[65]
93 (59)	n.d.	n.d.	Mixed	↑ CLD	Highest in Child-Pugh B	[72]
36 (64)	53.0 ± 1.3 (SEM)	27.1 ± 1.2 (SEM)	HCV	↑ HC	n.d.	[73]

**Table 2:** Adipokine levels in systemic venous serum (SVS), hepatic venous serum (HVS) and portal vein (PVS) blood of different cohorts with liver cirrhosis. Similar levels of adiponectin in those compartments were described in two studies [59,74]. There is one report showing splanchnic extraction of this adipokine, but levels in the different compartments were not presented [63]. Therefore, this study is not listed. n.d., not described; ≈ indicates similar levels in those compartments; ↑ indicates increased levels compared to the other compartments labeled with the identical upper case number (a, b); Interleukin6 (IL-6).

Adipokine	Cohort Size (% Male)	Age (Years)	BMI (kg/m^2^)	Etiology	SVS	HVS	PVS	References
Adiponectin	11 (n.d.)	n.d.	n.d.	Viral	≈	≈	≈	[59]
Adiponectin	50 (78)	55 ± 2	25.5 ± 0.9	Mostly alcoholic	≈	≈	≈	[74]
Chemerin	45 (80)	54 (26–81)	n.d.	Mostly alcoholic	≈	↑ ^a^	≈ ^a^	[83]
Leptin	50 (78)	55 ± 2	25.5 ± 0.9	Mostly alcoholic	↑ ^a^	≈ ^a^	≈ ^a^	[74]
Omentin	40 (80)	52 (26–81)	26.0 (16–38)	Mostly alcoholic	≈	≈ ^a^	↑ ^a^	[84]
Resistin	50 (78)	55 ± 2	25.5 ± 0.9	Mostly alcoholic	≈	≈	≈	[74]
Galectin-3	33 (75.8)	49 (40–81)	25.6 (17–38)	Alcoholic	≈	↑ ^a^	≈ ^a^	[85]
Visfatin	50 (78)	55 ± 2	25.5 ± 0.9	Mostly alcoholic	≈ ^a,b^	↑ ^a^	↑ ^b^	[74]
IL-6	41 (78)	n.d.	n.d.	Mostly alcoholic	↑ ^a,b^	≈ ^a,b^	↑↑ ^a^	[86]

**Table 3:** Chemerin levels in patients of two cohorts with liver cirrhosis. Number of patients, % males, age, body mass index (BMI) and disease etiology are listed. Numbers are given as median and range. Studies did not compare systemic levels of patients and respective control cohorts. The table lists associations of systemic chemerin with the Child-Pugh score. n.d., not described; HCV, hepatitis C virus.

Number of Patients (% Male)	Age (Years)	BMI (kg/m^2^)	Etiology	Systemic Levels	Negative Associations with Scores	Reference
44 (66)	71 (50–82)	22.5 (15.6–33.5)	Mostly HCV	n.d.	Child-Pugh	[103]
45 (80)	54 (26–81)	n.d.	Mostly alcohol	n.d.	Child-Pugh	[83]

**Table 4:** Leptin levels in different cohorts of patients with liver cirrhosis. Cohort size, % males, age, body mass index (BMI) and disease etiology are listed. Numbers are given as the mean ± standard deviation, or the standard errors of the mean (SEM), or as the median and range. ↑ indicates higher and ↓ lower systemic levels compared to the controls, and all studies enrolled healthy control cohorts. The table also lists associations of serum leptin with the Child-Pugh score. n.d., not described; HBV, hepatitis B virus; HCV, hepatitis C virus.

Number of Patients (% Male)	Age (Years)	BMI (kg/m^2^)	Etiology	Systemic Levels	Associations with Score	Reference
107 (100)	49.8 ± 11.5	n.d.	Alcohol	Unchanged	No	[60]
24 (66)	64 (64–75)	21.3 (17.3–26.9)	HCV	Unchanged	No	[115]
13 (0)	56.1 ± 7.4	22.6 ± 4.4	Alcohol	Unchanged	No	[119]
54 (56)	55.5 (38.3–72.8)	26.6 (20.6–32.7)	Mixed	n.d.	Child-Pugh negative	[118]
36 (64)	53.0 ± 1.3 (SEM)	27.11 ± 1.18 (SEM)	HCV	Unchanged	n.d.	[73]
40 (55)	57 ± 11	n.d.	Mixed	Unchanged	No	[114]
18 (100)	45 *±* 1.5 (SEM)	24.2 *±* 0.8 (SEM)	Alcohol	Unchanged	No	[112]
40 (88)	59 (37–75)	25.89 (20.1–39.3)	Alcohol	n.d.	No	[65]
26 (100)	59 (47–72)	23.7 (21.9–25.5)	Mixed	↑	No	[116]
10 (0)	44 *±* 3.7 (SEM)	26.4 *±* 2.2 (SEM)	Alcohol	↑	No	[112]
35 (49)	53 (28–73)	24 (18–33)	HBV/HCV	↑	No	[111]
24 (100)	51.7 ± 10.6	22.3 ± 4.0	Alcohol	↑	Child-Pugh positive	[119]
24 (48)	45.5 *±* 8.0	n.d.	HBV, HDV	↓	No	[113]
40 (0)	48.8 *±* 9.9	n.d.	Alcohol	↓	No	[60]

**Table 5:** Omentin levels in the patients of two cohorts with liver cirrhosis. Number of patients, % males, age, body mass index (BMI) and disease etiology are listed. Numbers are given as median and range. ↑ indicates higher systemic levels compared to the healthy controls (HC). There are no associations of systemic omentin with Child-Pugh and MELD scores. n.d., not described.

Number of Patients (% Male)	Age (Years)	BMI (kg/m^2^)	Etiology	Systemic Levels	Associations with Scores	Reference
40 (80.0)	52 (26–81)	26 (16–38)	Mostly alcohol	↑ HC trend	No	[84]
51 (68.6)	52 (26–80)	27 (17–40)	Mixed	n.d.	No	[132]

**Table 6:** Galectin-3 levels in patients of three cohorts with liver cirrhosis. Cohort size, % males, age, body mass index (BMI) and disease etiology are listed. Numbers are given as the mean ± standard deviation or as the median and range. ↑ indicates higher systemic galectin-3 levels compared to the controls. The table also lists associations of serum galectin-3 with Child-Pugh and model for end-stage liver disease (MELD) scores. n.d., not described; HC, healthy-liver controls; HBV, hepatitis B virus; HCV, hepatitis C virus; CLD, non-cirrhotic chronic liver disease.

Number of Patients (% Male)	Age (Years)	BMI (kg/m^2^)	Etiology	Serum Levels	Positive Associations with Scores	Reference
87 (n.d.)	n.d.	n.d.	Mostly alcohol	↑, HC Unchanged CLD	Child-Pugh	[141]
22 (55)	63.5 ± 10.8	n.d.	HBV/HCV	↑, CLD	n.d.	[142]
33 (76)	49 (40–81)	25.6 (17–38)	Alcohol	↑, HC	Child-Pugh MELD	[85]

**Table 7:** Resistin levels in six cohorts of patients with liver cirrhosis. Cohort size, % males, age, body mass index (BMI) and disease etiology are listed. Numbers are given as the mean ± standard deviation (not indicated), or the standard error of the mean (SEM), or as the median and range. ↑ indicates higher systemic levels compared to the controls. The table also lists associations of serum resistin with the Child-Pugh score. n.d., not described; HC, healthy controls; CLD, non-cirrhotic chronic liver disease; hepatitis B virus, HBV.

Number of Patients (% Male)	Age (Years)	BMI (kg/m^2^)	Etiology	Serum Levels	Positive Associations	Reference
147 (72.8)	49.6 ± 11.9	n.d.	Alcohol	↑ HC	No	[60]
79 (59.5)	66.6 ± 8.9	22.3 ± 2.9	HCV	↑ HC	Child-Pugh	[58]
40 (87.5)	59 (37–75)	25.9 (20.1–39.3)	Alcohol	n.d.	No	[65]
57	47.5 ± 1.3 (SEM)	23.0 ± 0.4 (SEM)	Mixed	↑ HC	Child-Pugh	[152]
50 (78)	55 ± 3	25.5 ± 0.9	Mostly alcohol	↑ HC	Child-Pugh	[74]
70 (60)	48.3 ± 11.1	n.d.	HBV	↑ HC, ↑ CLD	n.d.	[153]

**Table 8:** Visfatin levels in patients of five cohorts with liver cirrhosis. Cohort size, % males, age, body mass index (BMI) and disease etiology are listed. Numbers are given as the mean ± standard deviation, or the standard error of the mean (SEM), or as the median and range. ↑ indicates higher and ↓ lower systemic levels compared to the controls. The table also lists associations of serum visfatin with the Child-Pugh score. n.d., not described; HC, healthy controls; CLD, non-cirrhotic chronic liver disease; HBV, hepatitis B virus.

Number of Patients (% Male)	Age (Years)	BMI (kg/m^2^)	Etiology	Serum Levels	Associations	Reference
19 (68)	47.4 ± 2.5 (SEM)	23.0 ± 0.6 (SEM)	Mixed	↓ HC	No	[164]
40 (87.5)	59 (37–75)	25.89 (20.08–39.31)	Alcoholic	n.d.	No	[65]
129 (77.5)	48.71 ± 11.65	22.86 ± 3.89	HBV	↑ HC, ↑ CLD	n.d.	[165]
50 (78)	55 ± 3	25.5 ± 0.9	Mostly alcoholic	↓ HC	Child-Pugh positive	[74]
153 (59.5)	42–60	n.d.	HBV	↑ HC	n.d.	[166]

**Table 9:** IL-6 levels in patients of 10 cohorts with liver cirrhosis. Cohort size, % males, age, body mass index (BMI) and disease etiology are listed. Numbers are given as the mean ± standard deviation, or the standard error of the mean (SEM), or as the median and range. ↑ indicates higher systemic levels compared to the controls. The table also lists associations of serum IL-6 with the Child-Pugh or model for end-stage liver disease (MELD) score. n.d., not described; HC, healthy-controls; CLD, non-cirrhotic chronic liver disease; HBV, hepatitis B virus; HCV, hepatitis C virus.

Number of Patients (% Male)	Age (Years)	BMI (kg/m^2^)	Etiology	Serum Levels	Positive Associations in Patients	Reference
41 (56)	55 (40–81)	n.d.	Mixed	n.d.	Child-Pugh (trend)	[86]
43 (63)	56 (32–76)	n.d.	Alcohol	↑ HC	Child-Pugh (trend)	[177]
41 (63)	58 (29–74)	25.5 ± 0.9	HCV	No change, HC	No	[177]
70 (60)	48.3 ± 11.1	n.d.	HBV	↑ HC, ↑ CLD	n.d.	[153]
111 (60)	46 (18–70)	n.d.	Mixed	↑ HC, ↑ CLD	Child-Pugh	[40]
79 (62)	54.7 ± 14.7			↑ HC	n.d.	[174]
72 (67)	56.6 (36–75)	n.d.	HCV	↑ HC	Child-Pugh (trend)	[39]
72 (71)	53 (46–60)		Mixed	No change, HC	Child-Pugh	[183]
45 (38)	n.d.	n.d.	Alcohol	↑ (trend) HC	Child-Pugh (trend)	[175]
14 (78.6)	54 (44–59)	n.d.	Mostly HCV	↑ HC	MELD	[184]
